# MOSFET-Oriented Current Sharing Control Strategy for Scalable Parallel DC/DC Converters

**DOI:** 10.3390/mi17070818

**Published:** 2026-07-07

**Authors:** Mingzhe Qu, Yuan Zhou, Zhigang Zhang, Liangxing Hu, Yu Zhang

**Affiliations:** 1 School of Intelligent and Architectural Engineering, Harbin University, Harbin 150086, China; 2 College of Electrical Engineering, Harbin University of Science and Technology, Harbin 150080, China; 3 School of Electrical and Electronic Engineering, Nanyang Technological University, Singapore 639798, Singapore; 4 North Night Vision Technology Co., Ltd., Kunming 650217, China; 5 Kunming Institute of Physics, Kunming 650223, China

**Keywords:** scalable parallel converter system, phase-shifted full-bridge converter, MOSFET-oriented parallel current sharing

## Abstract

Parallel DC/DC converter modules provide a feasible approach for achieving power scalability in various power conversion systems. This paper investigates an MOSFET-based lagging leg series diodes phase-shift full-bridge (LLSD-PSFB) converter and proposes a three-loop current-sharing control strategy for coordinated parallel operation. The strategy incorporates a voltage loop, a current loop, and a current-sharing loop to mitigate load current imbalance caused by MOSFET parameter mismatches and module inconsistencies. The operating principle and parameter design of the single-module LLSD-PSFB converter are analyzed, and an averaged model is established. Based on this model, a small-signal model of the parallel system is derived to evaluate system stability and current-sharing performance. Simulation results demonstrate that the proposed control scheme effectively improves current-sharing accuracy and dynamic response. An experimental prototype is developed to validate the theoretical and simulation results. The experimental results confirm that the proposed three-loop control strategy achieves high current-sharing precision and stable operation, demonstrating its effectiveness for parallel DC/DC converter systems and its potential for scalable high-power applications.

## 1. Introduction

With the rapid growth of electric vehicles, scalable EV charging architectures based on parallel DC/DC converter modules have attracted increasing attention due to the demand for efficient and reliable charging infrastructure [1]. In practical applications, high output power is generally realized by paralleling multiple switched-mode DC/DC converter modules rather than employing a single centralized converter [2]. Compared with centralized architectures, parallel systems provide advantages in modularity, thermal management, redundancy, and scalability. However, parameter mismatches among parallel modules often result in current imbalance, which may lead to module overloading and degraded system reliability [3,4]. Therefore, effective current-sharing control has become a critical issue in scalable parallel converter systems.

Early current-sharing schemes for parallel converters were mainly based on analog control techniques [5]. Although these methods provide fast response and simple implementation, their performance is easily affected by component tolerances, temperature variations, and electromagnetic interference. Recent advances in digital control have enabled more flexible and robust current-sharing strategies for parallel converter systems [6,7,8]. The phase-shifted full-bridge (PSFB) converter [9,10,11] is widely used in medium- and high-power applications because of its simple structure and soft-switching capability. Nevertheless, several drawbacks remain, including circulating current, duty-cycle loss caused by transformer leakage inductance, and the loss of lagging-leg ZVS under light-load conditions. To overcome these problems, diode-assisted lagging-leg PSFB converters have been proposed to extend the ZVS range of the lagging-leg switches [12,13].

However, the additional series diodes introduce extra conduction losses. Auxiliary-resonant PSFB converters [14,15] further extend the soft-switching range by employing auxiliary resonant branches, but at the expense of increased circuit complexity and additional resonant losses. ZVZCS-based converters [16,17] can reduce switching losses through ZVS turn-on and ZCS turn-off; however, they generally require auxiliary circuits and more complex control schemes, leading to higher cost and increased design complexity. Most existing PSFB-based topologies mainly focus on extending the soft-switching range and reducing switching losses, whereas current-sharing performance in parallel scalable charging systems has received less attention [18,19].

To address this issue, a lagging-leg series-diode phase-shifted full-bridge (LLSD-PSFB) converter together with a triple-loop control strategy is proposed. The control scheme consists of a voltage loop, a current loop, and a current-sharing loop, enabling accurate current sharing among parallel modules. The proposed method maintains the soft-switching characteristics of the LLSD-PSFB converter while improving current-sharing accuracy and dynamic performance under parameter mismatches and load variations.

Current-sharing control has been widely investigated using methods such as droop control, master-slave control, average current-sharing control, and digital current-sharing control [20,21,22,23,24,25,26]. Although these approaches can achieve current balancing under specific operating conditions, their performance may degrade because of output-voltage deviation, communication dependence, increased implementation complexity, or limited robustness against parameter mismatches and load disturbances. These challenges motivate the development of the proposed triple-loop current-sharing strategy.

Therefore, to address the above challenges, this paper proposes a current-sharing control scheme for parallel DC/DC converters in scalable charging systems. This study is conducted on a lagging-leg series-diode phase-shifted full-bridge (LLSD-PSFB) converter, in which a triple closed-loop control strategy comprising voltage, current, and current-sharing loops is implemented. By integrating the current-sharing loop into the overall control framework, the proposed method improves current-sharing accuracy, enhances dynamic response performance, and strengthens system stability under parameter mismatch and load variation conditions, thereby improving the reliability and engineering applicability of scalable charging systems. In summary, the main contributions of this paper are as follows:

(1) The LLSD-PSFB topology utilizes the modulation effect of the series diodes on the lagging-leg current path to improve soft-switching performance over a wide load range. The proposed topology facilitates soft-switching operation of the switching devices, including ZVS and ZCS characteristics, which helps reduce switching losses and current stress.
(2) Given the modeling complexity of the LLSD-PSFB topology arising from the nonlinear switching behavior and soft-switching dynamics of the MOSFET devices, an averaged switching modeling approach is adopted in this study. The switching process of the main power stage, particularly the MOSFET commutation intervals and energy transfer mechanisms, is equivalently linearized, leading to a comprehensive small-signal AC model. Furthermore, the key transfer functions are analytically derived to characterize the dynamic response and switching stress of the MOSFETs.
(3) The performances of the proposed control strategy are verified through both simulation and experimental results. A detailed comparison between theoretical analysis and simulation outcomes is conducted to validate the structural design, particularly from the perspective of MOSFET switching behavior and stress distribution. Furthermore, the effectiveness of the proposed current-sharing mechanism is confirmed by demonstrating balanced current distribution and reduced switching stress among the MOSFET devices.

The remainder of this paper is organized as follows: Section 2 describes the system configuration. Section 3 elucidates the operational principles of the topology, the conditions for achieving zero-voltage and zero-current switching (ZVZCS), as well as the advantages and disadvantages of the topology. Additionally, it introduces the lagging leg series diodes phase shift full bridge converter (LLSD-PSFB). Section 4 provides a small-signal modeling and analysis of the LLSD-PSFB. Section 5 presents simulation and experimental results to validate the effectiveness of the proposed control strategy. The conclusion summarizes the main findings of this study and highlights key directions for future research.

## 2. System Architecture Design

### 2.1. The Basic Structure of the System

**Figure 1:**
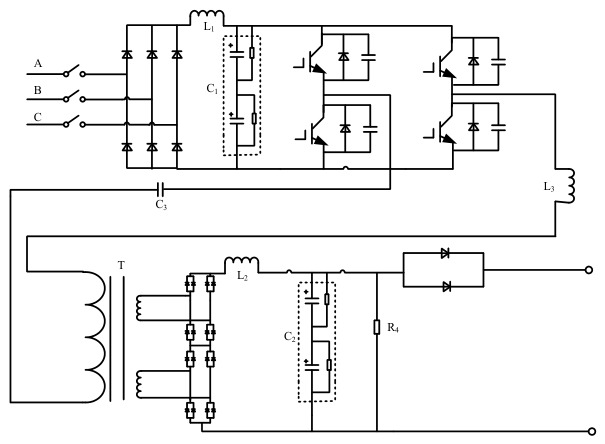
Main circuit schematic diagram.

The fundamental principles of scalable charging systems based on parallel DC/DC converter modules are illustrated in Figure 1. Alternating current from the power grid is rectified into direct current voltage via a rectification circuit. This direct current is then processed through an LC filter circuit to produce the direct current voltage required for the input stage of the DC/DC converter. Subsequently, the voltage undergoes chopping and transformation, is coupled through a transformer, and is finally rectified and filtered to yield the desired direct current output voltage and current.

The switching frequency of DC/DC converters is typically chosen within 20–150 kHz. Considering the trade-off between audible noise, switching losses, and system cost, a frequency of 40 kHz is selected. The proposed LLSD-PSFB converter is designed for a 48 V DC input, a regulated 14 V output (±0.5 V), and a rated power of 2 × 300 W.

### 2.2. Conventional Soft-Switching Phase-Shifted Full-Bridge Converter Topology

#### 2.2.1. Zero-Voltage-Switching Phase-Shifted Full-Bridge Converter

Zero-voltage switching (ZVS) can be achieved by utilizing the leakage inductance of the transformer primary winding or an additional series inductance together with a capacitor connected in parallel with the switch. Before the switch is turned on, the voltage across the parallel capacitor is discharged to zero, ensuring that the switch voltage is zero at the instant of turn-on. Although ZVS is achieved during the turn-on process, the proposed converter mainly focuses on achieving zero-current turn-off (ZCS) for the MOSFET switches.

**Figure 2:**
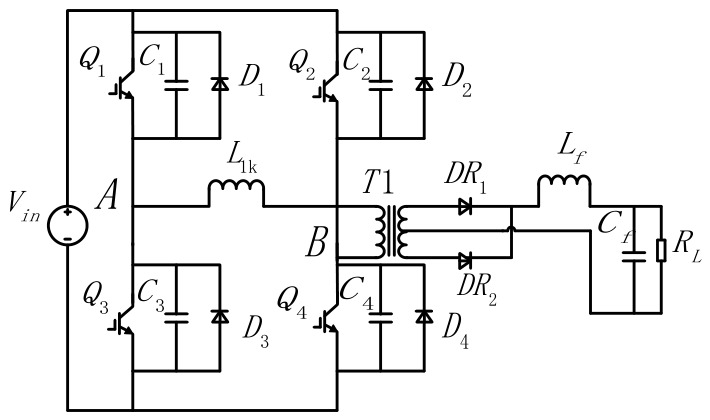
ZVS phase-shifted full-bridge converter topology.

Figure 2 shows the circuit topology of the proposed phase-shifted full-bridge converter, where the four switches are controlled using phase-shift modulation. For theoretical analysis, several simplifying assumptions are adopted. All circuit components are considered ideal, and the transformer turns ratio is defined as K.

#### 2.2.2. ZCS Phase-Shifted Full-Bridge Converter Topology

**Figure 3:**
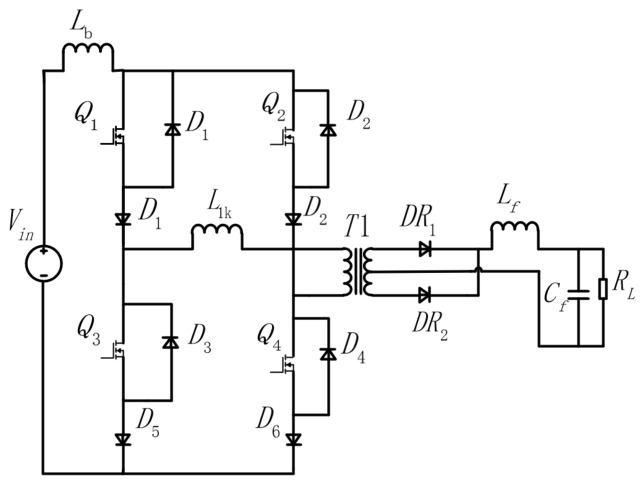
ZCS phase shift full bridge converter topology.

Figure 3 depicts a typical ZCS circuit configuration. Although phase-shift control is also adopted, the switching sequence differs from that of the previously described ZVS circuit. Specifically, the turn-on of switch *Q*_4_ precedes that of *Q*_1_, while *Q*_3_ is activated prior to *Q*_2_. Moreover, the two switches within the same bridge leg do not operate in a complementary manner; instead, a finite overlap exists between their conduction intervals.

The operating conditions of the ZCS phase-shifted full-bridge converter are more demanding than those of the ZVS phase-shifted full-bridge converter, necessitating greater voltage and current ratings for the power switching devices. Due to the presence of numerous diodes in the ZCS phase-shifted full-bridge converter circuit, and considering the reverse blocking characteristics of these diodes, they must withstand high reverse voltage levels.

## 3. Proposed Phase-Shifted Full-Bridge Converter

### 3.1. Zero-Voltage and Zero-Current Switching Phase-Shifted Full-Bridge Converter

Analysis of the operating principles of the ZVS and zero-current switching (ZCS) phase-shifted full-bridge converters indicates that achieving ZVS in the leading leg is challenging, whereas ZCS is more readily attainable. Consequently, this study employs a ZVZCS phase-shifted full-bridge converter.

**Figure 4:**
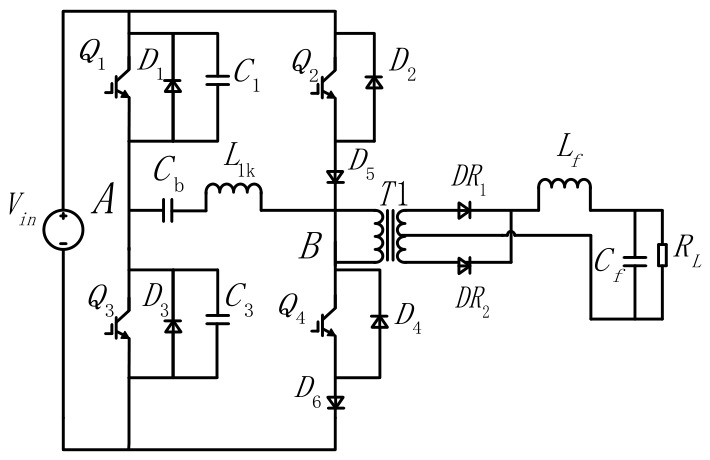
Main circuit of the LLSD-PSFB converter.

By integrating the advantages of the aforementioned ZVS and ZCS phase-shifted full-bridge converter topologies, the front leg is designed as a ZVS phase-shifted full-bridge structure. Additionally, a diode is connected in series beneath each of the two switching devices in the rear leg, facilitating ZCS. The topology is illustrated in Figure 4. The purpose of the series-connected diodes is to prevent the reverse rise in the primary-side current, thereby clamping the current at zero for a brief period to achieve zero-current turn-on. Although the series-connected diodes in the rear leg introduce conduction losses, these losses remain relatively minor compared to the energy savings realized through zero-current switching. Therefore, this topology is not only structurally simple but also exhibits excellent overall performance.

Based on the analysis presented, this paper proposes a topological structure known as the lagging leg series diode phase-shifted full-bridge converter (LLSD-PSFB). This configuration achieves zero-voltage zero-current switching (ZVZCS), thereby reducing switching losses. Furthermore, the structure is relatively simple, which not only reduces costs but also accelerates deployment speed; it requires the addition of only two diodes to facilitate zero-current switching. The parameters associated with the proposed topology are summarized in Table 1.

### 3.2. Operating Principle of the LLSD-PSFB Converter

In the LLSD-PSFB topology, a diode is inserted in series with both the upper and lower switches of the lagging leg. This series diode suppresses the reverse increase in the primary-side current, thereby clamping the current to zero for a finite interval. As a result, zero-current switching (ZCS) is achieved for the two power switches in the lagging leg.

**Figure 5:**
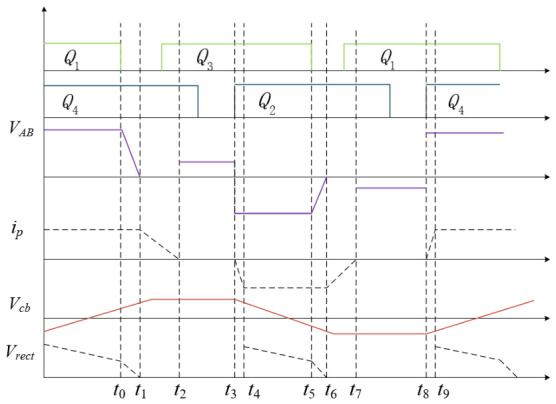
Key waveforms of the main circuit.

A detailed analysis of the operating principle of the LLSD-PSFB converter is presented as follows. Figure 4 shows the main circuit configuration, and Figure 5 illustrates the corresponding key waveforms. From top to bottom, the illustrated signals include the conduction states of the leading-leg switches *Q*_1_ and *Q*_3_, the conduction states of the lagging-leg switches *Q*_2_ and *Q*_4_, the bridge voltage *U*_*AB*_, the primary-side current *i*_*p*_, the blocking capacitor voltage *V*_*Cb*_, and the transformer secondary voltage *V*_*rect*_.

For the purpose of simplifying the analysis, the following assumptions are adopted. All circuit components are considered ideal. The relationship $K^{2}L_{f}\gg L_{lk}$ is satisfied, where K denotes the transformer turns ratio. The resonant capacitors satisfy $C_{1}=C_{3}=C_{r}$. In addition, the blocking capacitor $C_{b}\;$ is assumed to be sufficiently large so that its voltage can be regarded as approximately constant during one switching period.

**Figure 6:**
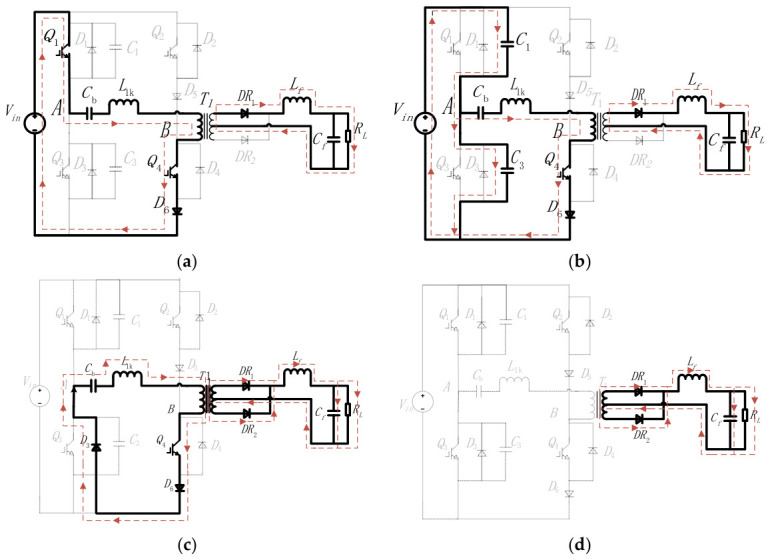

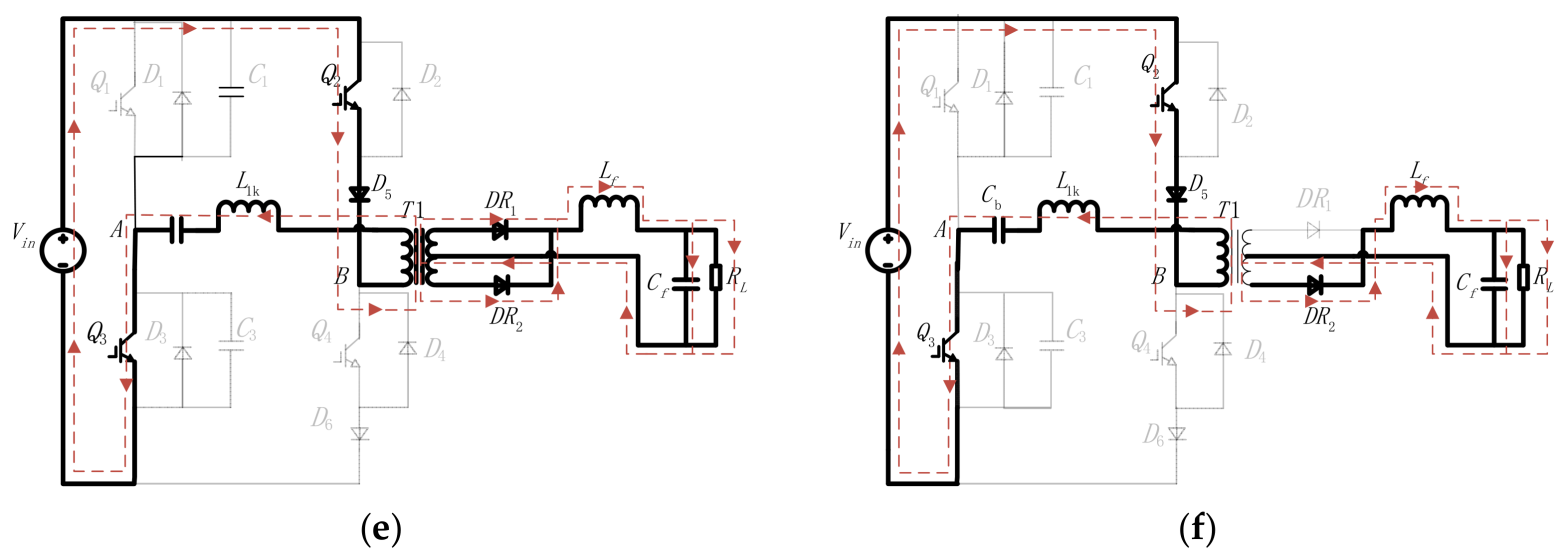
Current flow paths of the LLSD-PSFB main circuit during each operating interval. (**a**) [0–*t*_0_]; (**b**) [*t*_0_–*t*_1_]; (**c**) [*t*_1_–*t*_2_]; (**d**) [*t*_2_–*t*_3_]; (**e**) [*t*_3_–*t*_4_]; (**f**) [*t*_4_–*t*_5_].

At *t*_0_, switches *Q*_1_ and *Q*_4_ are in the on-state. The primary-side current flows through *Q*_1_, the blocking capacitor *C_b_*, the leakage inductor *L*_lk_, the transformer primary winding *Q*_4_, and diode *D*_6_, before returning to the negative terminal of the DC source. The corresponding current path is illustrated in Figure 6a. During the interval from *t*_0_ to *t*_1_, *Q*_1_ is in the zero-voltage turn-off state, while *Q*_4_ remains conducting. On the secondary side, diode DR_1_ conducts, and DR_2_ is reverse-biased. The secondary-side inductance, reflected to the primary side, combines with the primary leakage inductance to form the equivalent resonant inductance. This resonant inductance, together with the external inductance, resonates with capacitors $C_{1}$ and $C_{3}$. As a result, $C_{1}$ is charged, and its voltage increases linearly, whereas the voltage across $C_{3}$ decreases linearly to zero. Because the resonant inductance is relatively large, the current variation during this interval is negligible. Therefore, the primary-side current can be approximated as a constant current source with a magnitude of $I_{o}/K$. Meanwhile, the blocking capacitor $C_{b}$ continues charging, and its voltage $V_{Cb}$ increases accordingly. The corresponding current path from *t*_0_ to *t*_1_ is shown in Figure 6b.

(1) $$V_{\mathrm{cb}}(t)=V_{\mathrm{cb}}\left(t_{0}\right)+I_{p0}\times \frac{t-t_{0}}{C_{b}}$$

(2) $$u_{\mathrm{cl}}(t)=\frac{I_{p0}}{C_{1}+C_{3}}t=\frac{I_{p0}}{2C_{r}}t$$

(3) $$u_{c3}(t)=V_{\mathrm{in}}-\frac{I_{p0}}{2C_{r}}t$$

At *t*_1_, the resonance process is completed, and the voltage across *C*_3_ decreases to zero. The duration from *t*_0_ to *t*_1_ is expressed as follows. At $t_{1}$, the resonant transition is completed, and the voltage across $C_{3}$ decreases to zero. The time interval *t*_0_ to *t*_1_ can be expressed as

(4) $$t_{01}=2C_{r}V_{\mathrm{in}}/I_{p0}$$

By substituting (4) into (1), we obtain:

(5) $$V_{\mathrm{cb}}\left(t_{1}\right)=V_{\mathrm{cb}}\left(t_{0}\right)+2\times \frac{C_{r}V_{\mathrm{in}}}{C_{b}}$$

During the interval *t*_1_ to *t*_2_, *Q*_3_ can be turned on only after the voltage across $C_{3}\;$ has fallen to zero. Therefore, the dead time $t_{d(lead)}$ between the driving signals of *Q*_1_ and *Q*_3_ must exceed the discharge duration of $C_{3}$, i.e., $t_{01}=t_{1}-t_{0}$. Accordingly, the following condition should be satisfied:

(6) $$t_{d(lead)}>2C_{r}V_{in}\;/\;I_{p0}$$

Figure 6c shows the current path during this interval, where the bold lines indicate the conduction loop. The primary-side current decreases gradually while maintaining the same direction, causing the blocking capacitor $C_{b}$ to continue charging and its voltage $V_{Cb}\;$ to increase at a reduced rate. Meanwhile, both secondary-side diodes *DR*_1_ and *DR*_2_ conduct simultaneously, effectively short-circuiting the transformer secondary and clamping the transformer terminal voltage to zero. Consequently, the voltage across $C_{b}$ is applied to the leakage inductance. Given the small leakage inductance and the large capacitance of $C_{b}$, the blocking capacitor can be approximated as a constant voltage source during this interval. The expressions for $V_{Cb}$ and the primary current $i_{p}$ are given in (7) and (8), respectively.

(7) $$V_{\mathrm{cb}}(t)=V_{\mathrm{cb}}\left(t_{1}\right)=V_{\mathrm{cbp}}$$

(8) $$i_{p}(t)=I_{p0}-\frac{V_{\mathrm{cbp}}}{L_{1k}}t$$

During the interval *t*_2_ to *t*_3_, the secondary current is zero, and the bridge voltage between points A and B is therefore imposed across the blocking capacitor $C_{b}$, with a magnitude of $V_{Cbp}$. At this stage, both secondary-side diodes DR_1_ and DR_2_ conduct simultaneously, establishing the commutation condition for *Q*_4_. Consequently, *Q*_4_ turns off under zero-current conditions. The corresponding current path is shown in Figure 6d.

During the interval *t*_3_ to *t*_4_, the gate signal of *Q*_2_ is applied at $t_{3}$, when the primary current is zero. Owing to the presence of primary-side leakage inductance, the inductor current cannot change instantaneously; therefore, *Q*_2_ achieves zero-current switching at turn-on. Subsequently, the primary current increases gradually in the reverse direction. However, its magnitude is insufficient to transfer energy to the load, and thus the secondary-side diodes DR_1_ and DR_2_ remain conducting. The corresponding current path is shown in Figure 6e. The reverse current causes the blocking capacitor $C_{b}$ to discharge, leading to a linear decrease in its voltage $V_{Cbp}$. The voltage across the leakage inductance is given by $-(V_{in}+V_{Cbp})$; as $V_{Cbp}$ decreases, the inductor voltage correspondingly reduces. The expression of the primary current $i_{p}$ is provided in (9).

(9) $$i_{p}(t)=-\frac{V_{in}+V_{cbp}}{L_{lk}}t$$

At $t_{4}$, the primary current increases to $I_{o}/K$, which is sufficient to transfer energy to the load. Substituting $t=t_{4}$ and $i_{p}=I_{o}/K$ into (9), the duration $t_{34}=t_{4}-t_{3}$ can be obtained.

(10) $$t_{34}=\frac{L_{\mathrm{lk}}\times I_{p0}}{V_{\mathrm{in}}+V_{\mathrm{cbp}}}$$

During the interval t_4_ to t_5_, switches *Q*_2_ and *Q*_3_ conduct simultaneously, enabling energy transfer to the secondary side. This operating mode is analogous to that at $t_{0}$. The blocking capacitor $C_{b}$ initially discharges to zero and subsequently charges with reversed polarity. On the secondary side, *DR*_2_ conducts while *DR*_1_ is reverse-biased. The corresponding current path is shown in Figure 6f. The voltage across the blocking capacitor during this interval can be expressed as:

(11) $$V_{\mathrm{cb}}(t)=V_{\mathrm{cbp}}-\frac{I_{p0}}{C_{b}}t$$

At $t_{5}$, the voltage across the blocking capacitor $V_{Cb}$ is given by

(12) $$V_{cb}\left(t_{5}\right)=V_{cbp}-\frac{I_{p0}}{C_{b}}t_{45}$$

At $t_{5}$, switch *Q*_3_ is turned off. Before this instant, the parallel capacitor $C_{3}$ has been fully discharged to zero, enabling *Q*_3_ to achieve zero-current turn-off. The operation in the second half of the switching cycle is similar to that in the first half and is therefore omitted for brevity.

## 4. Small-Signal Modeling of the LLSD-PSFB Converter

### 4.1. LLSD-PSFB Small-Signal Modeling

**Figure 7:**
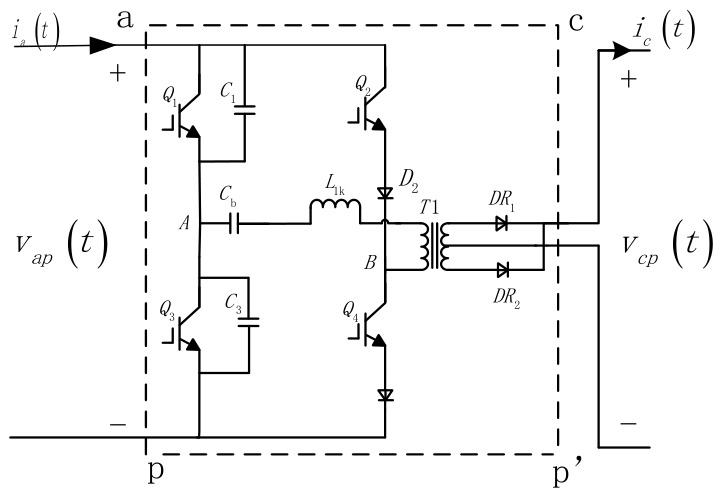
Switching network of the LLSD-PSFB.

The small-signal AC equivalent model derived using the averaged switch modeling method provides a more accurate representation of the converter dynamics. Accordingly, the resulting transfer function more accurately reflects the dynamic characteristics of the converter. Therefore, this method is adopted in this study to establish the small-signal model, laying a solid foundation for subsequent closed-loop control design. Figure 7 shows the switching network of the LLSD-PSFB converter.

The circuit enclosed by the dashed box, denoted as acp′p, represents a switching network. The current entering port a is defined as $i_{a}(t)$, and the current leaving port c is defined as $i_{c}(t)$. The voltage across ports a and *p* is denoted as $V_{ap}(t)$. Since nodes *p* and *p*′ serve as ground reference points, they are considered electrically identical. Therefore, the output voltage across ports c and *p*′ is expressed as $V_{cp}(t)$.

**Figure 8:**
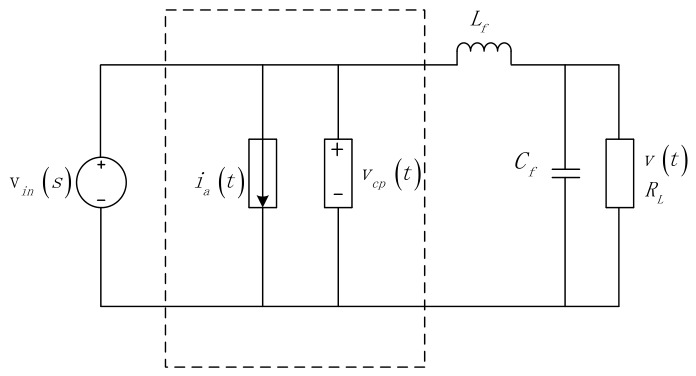
Controlled-source equivalent of the switching network.

By selecting $V_{ap}(t)$ and the output filter inductor current $i_{c}(t)$ as the input variables, the output variables of the switching network are defined as the output voltage $V_{cp}(t)\;$ and the input current $i_{a}(t)$. To preserve the port characteristics, the switching network is equivalently replaced by a controlled source, as illustrated in Figure 8.

**Figure 9:**
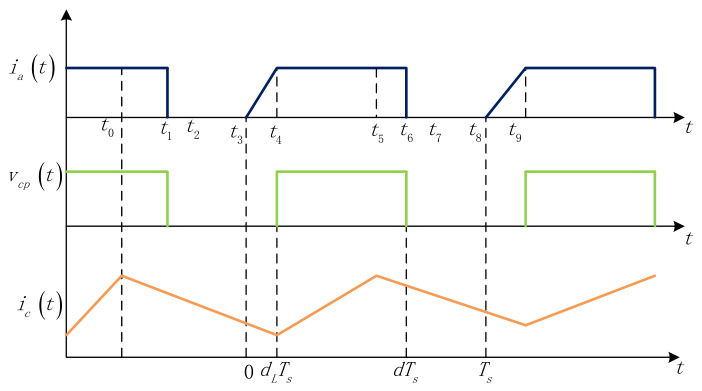
Port waveform of the equivalent controlled source.

Based on the operating principle of the LLSD-PSFB converter, the time-domain waveforms of the switching network variables can be derived. The waveform corresponds to that across the two ports of the equivalent controlled source, as shown in Figure 9, including $i_{a}(t)$, $V_{cp}(t)$, and $i_{c}(t)$.

Based on the switching network analysis, the state-space averaging method is applied and linearized around the steady-state operating point. The resulting small-signal transfer function $G_{vd}(s)$, which relates duty-cycle perturbations to output-voltage perturbations, is given in (13).

(13) $$\begin{array}{l} G_{\mathrm{vd}}(s)=\frac{{\hat{v}}_{o}(s)}{\hat{d}(s)}=\frac{\frac{V_{\mathrm{in}}\hat{d}(s)}{n}\frac{R_{L}//\frac{1}{sC}}{R_{L}//\frac{1}{sC}+sL+R_{d}}}{{\hat{d}}_{s}}\\ \;\;=\frac{U_{\mathrm{in}}/n}{s^{2}LC+s\left(L/R_{L}+R_{d}C\right)+1+R_{d}/R_{L}} \end{array}$$

(14) $$R_{d}=-\frac{V_{\mathrm{in}}}{n}G_{i}=-\frac{V_{\mathrm{in}}}{n}\times \left(-\frac{L_{\mathrm{lk}}}{nT_{s}\left(V_{\mathrm{in}}+V_{\mathrm{cbp}}\right)}\right)$$

where *n* is the transformer turns ratio between the primary and secondary windings, Ts denotes the switching period, d^^^(s) represents the Laplace-domain small-signal perturbation of the duty cycle, and R_d_ denotes the equivalent conduction resistance of the current path.

Similarly, the duty-cycle-to-inductor-current transfer function $G_{id}(s)$ is given in (15).

(15) $$\begin{array}{l} G_{\mathrm{id}}(s)=\frac{{\hat{i}}_{L}(s)}{\hat{d}(s)}=\frac{\frac{V_{\mathrm{in}}\hat{d}(s)/n}{s_{L}//\frac{1}{sC}+sL+R_{d}}}{\hat{d}(s)}\\ \;\;=\frac{\frac{V_{\mathrm{in}}}{n}\times \frac{1+sCR_{L}}{R_{L}}}{s^{2}LC+s\left(L/R_{L}+R_{d}C\right)+1+R_{d}/R_{L}} \end{array}$$

### 4.2. Current-Sharing Control of Parallel Converters Under Three-Loop Regulation

Compared with voltage-mode control, current-mode control provides improved dynamic response for individual switch-mode power supply modules. Its advantages include enhanced stability, inherent harmonic compensation, and strong disturbance rejection capability. Therefore, an average current-mode control scheme is implemented for a single module using the DSP28335 digital signal processor.

**Figure 10:**
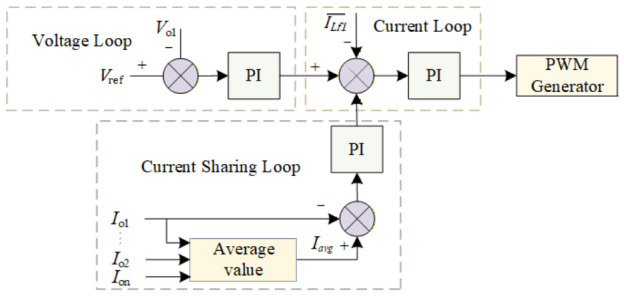
Schematic diagram of the three−loop current-sharing control strategy.

The average current-sharing method provides high current-sharing accuracy among parallel modules. A DSP-based digital control scheme is adopted, and current information is exchanged through communication links to achieve accurate current sharing. As a result, the current-sharing accuracy can be significantly improved. Accordingly, a digital current-sharing control scheme is implemented for the parallel system. Based on the above analysis, a three-loop current-sharing control strategy is adopted to achieve current equalization among the parallel modules. The corresponding control structure is shown in Figure 10.

**Figure 11:**
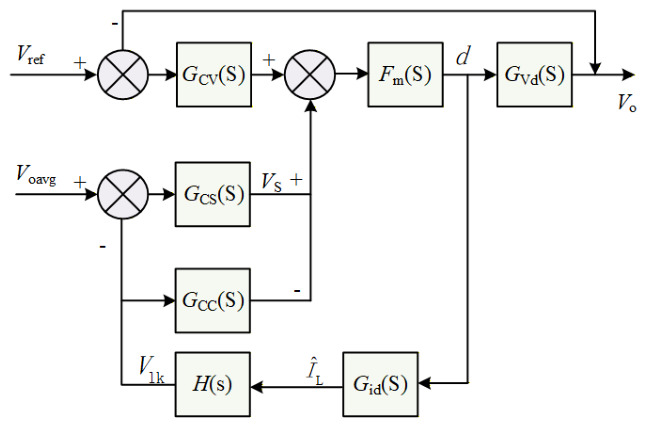
Block diagram of three−loop current sharing control strategy.

The operational principle of the three-loop current-sharing control strategy is described as follows. The output currents of the parallel modules are measured and fed into the current-sharing loop, where their average value is calculated. The difference between the average current and each module’s current is processed by a proportional-integral (PI) controller. The PI output, together with the voltage-loop output, is used as the reference signal for the current loop. The feedback signal of the current loop is the output inductor current of each module. Through this coordinated three-loop structure, the module currents gradually converge to the average value, thereby achieving accurate current sharing. By incorporating the small-signal AC equivalent model of the LLSD-PSFB converter, the overall control block diagram of the system can be established, as shown in Figure 11. The transfer functions are defined as follows: G_CV_(S), G_CC_(S), and G_CS_(S) denote the voltage loop, current loop, and current-sharing loop transfer functions, respectively. H(S) represents the current-to-voltage feedback transfer function; Fm(S) is the PWM modulator transfer function; G_Vd_(S) and G_id_(S) denote the duty-cycle-to-output-voltage and duty-cycle-to-inductor-current transfer functions, respectively.

Based on the control block diagram, the open-loop gain of the voltage loop can be derived as follows:

(16) $$T_{V}(s)=F_{m}(s)G_{\mathrm{cv}}(s)G_{\mathrm{vd}}(s)$$

The open-loop gain of the current loop is given by

(17) $$T_{i}(s)=F_{m}(s)G_{\mathrm{cc}}(s)H(s)G_{\mathrm{id}}(s)$$

The open-loop gain of the current-sharing loop can be obtained as

(18) $$T_{s}(s)=F_{m}(s)G_{\mathrm{cs}}(s)H(s)G_{\mathrm{id}}(s)$$

The overall output gain of the parallel system can be expressed as

(19) $$T(s)=\frac{T_{v}(s)}{1+T_{i}(s)+T_{s}(s)}$$

The parameters of the PI controllers used in the proposed triple closed-loop control strategy are listed in Table 2. The PI gains are obtained based on the controller design requirements and system dynamic characteristics. The zero frequencies of the controllers are also provided to describe the bandwidth characteristics of each control loop.

**Figure 12:**
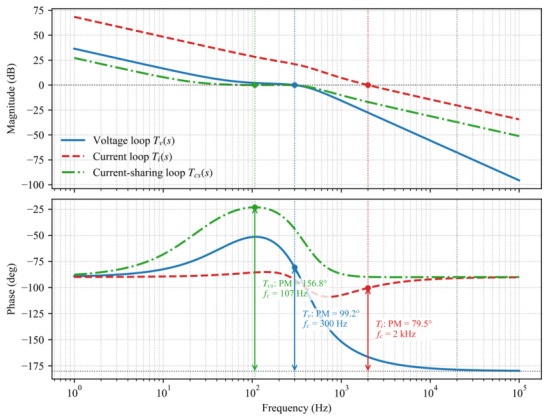
Bode plots of the proposed closed-loop control system.

To verify the stability and dynamic characteristics of the proposed triple closed-loop control strategy, frequency-domain analysis is performed based on the developed small-signal model. The frequency responses of the voltage loop, current loop, and current-sharing loop are shown in Figure 12.

As shown in the figure, the crossover frequencies of the three control loops are 107 Hz, 300 Hz, and 2 kHz, respectively, satisfying the design principle that the bandwidth of the inner loop should be higher than that of the outer loop. The corresponding phase margins are calculated as 156.8°, 99.2°, and 79.5°, all of which exceed the empirical stability threshold for practical engineering applications. All three control loops maintain high open-loop gain at low frequencies to effectively suppress steady-state static errors of the system, while their magnitude responses roll off continuously as frequency increases in the high-frequency region, enabling satisfactory attenuation against high-frequency disturbances.

## 5. Application Verification

To validate the comprehensive performance and parameter design rationale of the lagging leg series diodes phase-shift full-bridge (LLSD-PSFB) converter proposed in this study, we first conducted simulations of a single-module LLSD-PSFB. Subsequently, we constructed a prototype to verify the operational characteristics of the LLSD-PSFB and the current-sharing effectiveness of the three-loop current control.

### 5.1. Simulation Verification of a Single LLSD-PSFB Module

**Figure 13:**
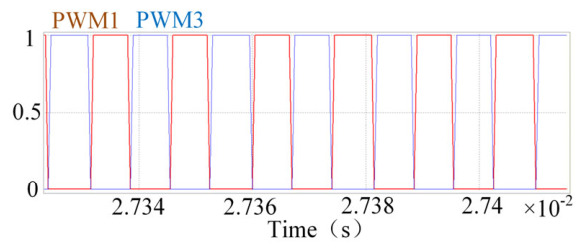
The control signal voltage of leading bridge arm MOSFET.

**Figure 14:**
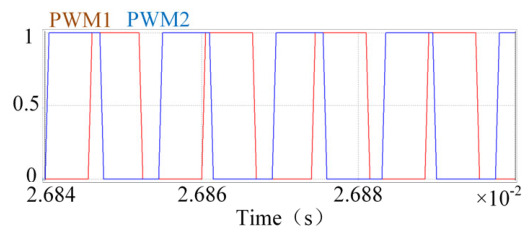
Control signal of the upper switch tube of two bridge arms.

In PSIM (trial version), the main circuit of the single-module ZVZCS phase-shifted full-bridge converter, together with the dual-loop control and phase-shifting circuits, was established. After the system reached steady-state operation, the control signals of the two leading-leg switches and the upper switches of both bridge arms are shown in Figure 13 and Figure 14, respectively. It is observed that the control signals exhibit the expected dynamic phase-shifting behavior, thereby verifying the correctness and effectiveness of the implemented phase-shift control circuit.

**Figure 15:**
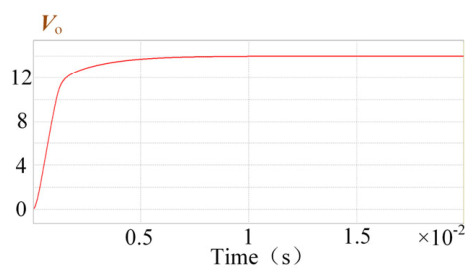
Output voltage waveform.

**Figure 16:**
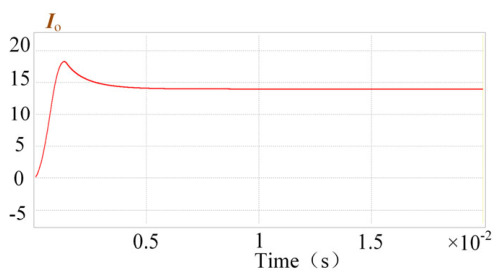
Output current waveform.

With an input DC voltage of 48 V and a load resistance of 1 Ω, the output voltage and current of the converter are shown in Figure 15 and Figure 16, respectively. In Figure 15, the output voltage increases smoothly from zero and settles at approximately 14 V after a short transient process, indicating effective voltage regulation capability. The corresponding output current in Figure 16 confirms stable converter operation under the specified operating condition.

**Figure 17:**
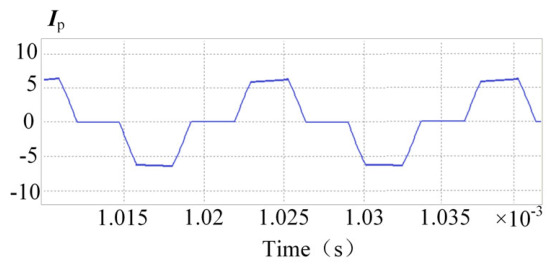
Converter primary side current.

**Figure 18:**
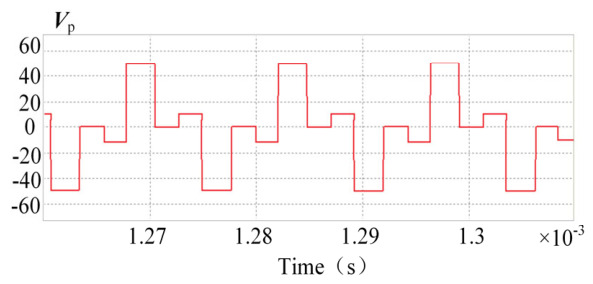
Primary side voltage of the converter.

The transformer primary-side current and voltage waveforms are shown in Figure 17 and Figure 18. In Figure 17, the primary-side current exhibits a stable periodic waveform under steady-state operation, indicating continuous energy transfer and stable converter operation. Figure 18 shows that the primary-side voltage presents the expected phase-shift modulation characteristics with regular voltage transitions during the switching process. These results verify the effectiveness of the proposed phase-shift control strategy and the correct operation of the converter topology.

**Figure 19:**
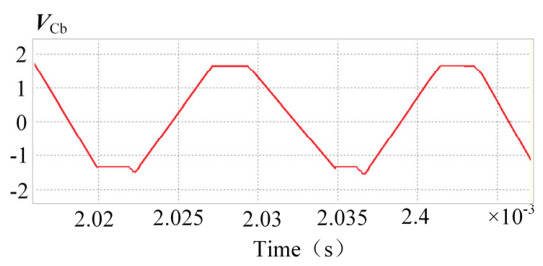
Output voltage waveform at both ends of the blocking capacitor.

**Figure 20:**
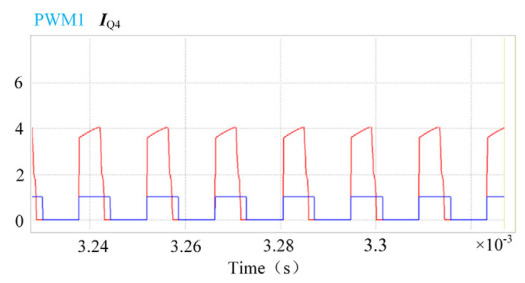
The zero current waveform of the MOSFET on the hysteresis bridge arm.

Figure 19 and Figure 20 present the blocking capacitor voltage and the MOSFET current of the hysteresis bridge arm. The blocking capacitor voltage shows a stable triangular waveform without noticeable voltage drift, indicating balanced charging and discharging during steady-state operation. The MOSFET current periodically decreases to zero prior to the switching transition, ensuring the condition for zero-current switching. The obtained waveforms agree with the theoretical analysis and demonstrate the normal operation of the converter.

**Figure 21:**
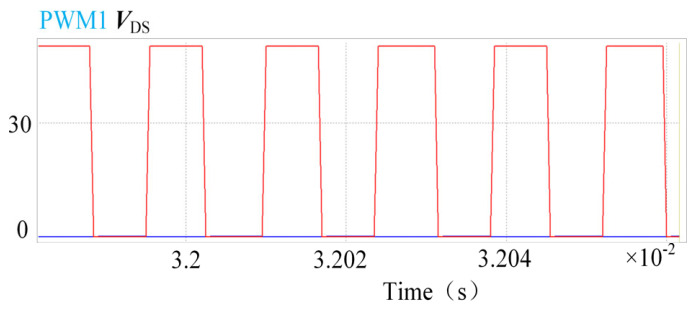
Zero-voltage waveform of the MOSFET on the leading bridge arm.

**Figure 22:**
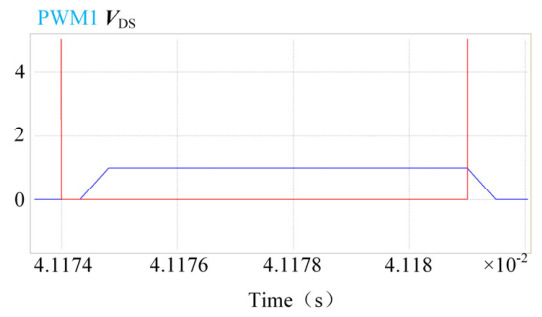
Detail enlarged view of zero-voltage waveform.

The zero-voltage waveform on the leading leg and the corresponding enlarged switching waveform are shown in Figure 21 and Figure 22, respectively. It can be observed that the voltage across the device decreases to nearly zero before turn-on. Consequently, the MOSFET is switched on under a zero-voltage condition, which contributes to reduced switching loss. The experimental results are consistent with the expected soft-switching operation.

### 5.2. Simulation Verification of Parallel LLSD-PSFB Converter

**Figure 23:**
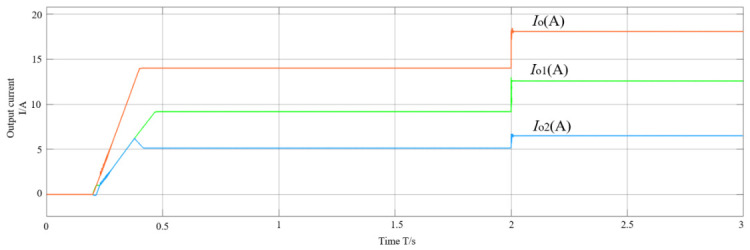
Output current waveform without current sharing measures.

In the absence of the current-sharing control strategy, the output currents of the parallel modules are shown in Figure 23. Before t = 2 s, the load resistance is set to 1 Ω. At 2 s, the load resistance is changed from 1 Ω to 0.75 Ω. It can be observed that the output currents of the parallel modules are significantly different under both steady-state and transient conditions, indicating that satisfactory current sharing cannot be achieved without an additional current-sharing control loop.

**Figure 24:**
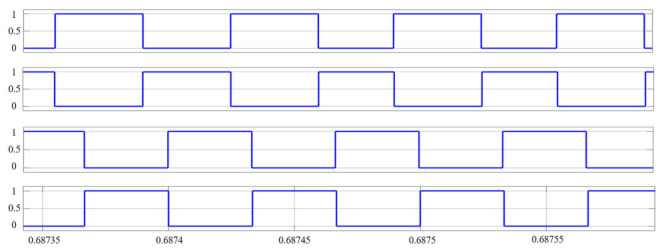
Four-channel PWM signal waveform.

With the implementation of the current-sharing control strategy, the PWM driving signals of one converter module are shown in Figure 24. Four gate signals are generated for the full-bridge switches. The switches in each bridge leg operate complementarily, while a phase-shift angle is introduced between the leading and lagging legs, matching the intended phase-shift modulation scheme.

**Figure 25:**
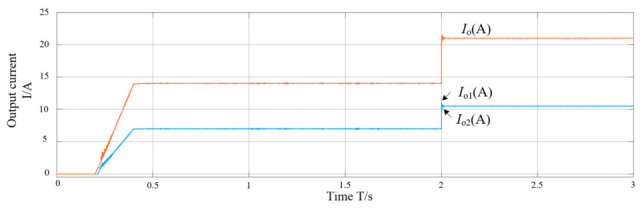
The waveform of the output current.

The output-current waveform obtained with the proposed triple-loop control strategy is presented in Figure 25. The currents delivered by the two converter modules exhibit nearly identical amplitudes over the entire operating range. After the load resistance changes, both module currents increase simultaneously and continue to share the load evenly. The total output current corresponds to the sum of the two module currents.

### 5.3. Experimental Verification of the Parallel LLSD-PSFB System

To verify the feasibility of the proposed LLSD-PSFB converter, a prototype was built based on the designed parameters. The control system is implemented using a DSP TMS320F28335. The switching frequency is set to 40 kHz, and the control variables are updated in each switching cycle.

**Figure 26:**
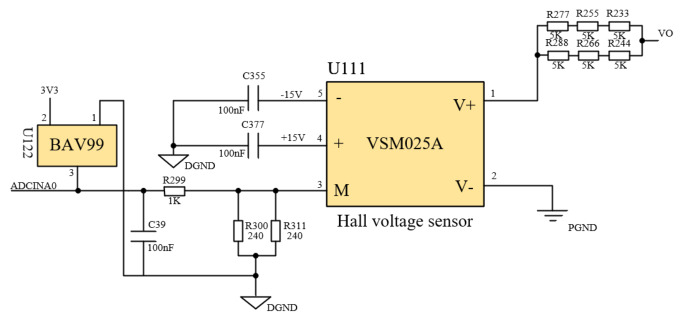
Voltage sampling circuit.

For voltage feedback in the closed-loop control system, a VSM025A closed-loop Hall voltage sensor is used to measure the output voltage. The voltage sampling circuit is presented in Figure 26. The sensor converts the high-voltage signal into a proportional secondary current through galvanic isolation, and the current signal is then converted into a voltage signal by a sampling resistor network. Afterward, the signal is filtered by an RC low-pass filter and limited by a diode clamping circuit. This circuit provides voltage isolation and noise filtering for accurate voltage acquisition.

**Figure 27:**
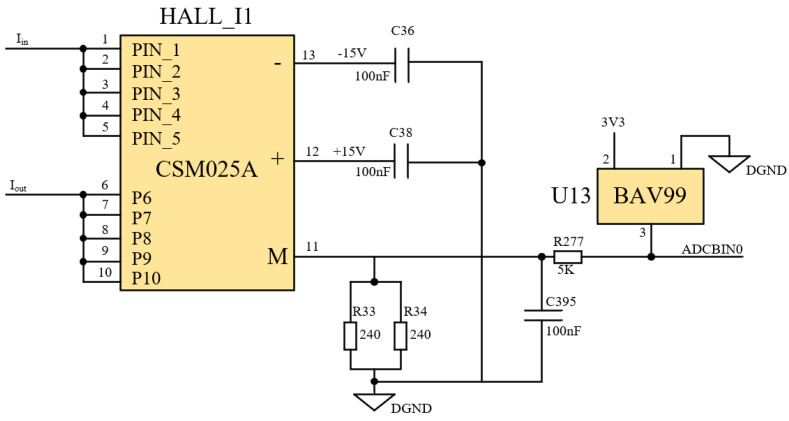
Current sampling circuit.

The output current and inductor current are measured using CSM025A Hall-effect current sensors with a primary-to-secondary ratio of 1:1000. The current sampling circuit is shown in Figure 27. The sensed current signals are converted into voltage signals through the sampling circuit, and then processed by an RC low-pass filter and a diode clamping circuit before being sampled by the DSP ADC module. The current sampling circuit provides signal isolation and conditioning, ensuring accurate current feedback for the closed-loop control system.

**Figure 28:**
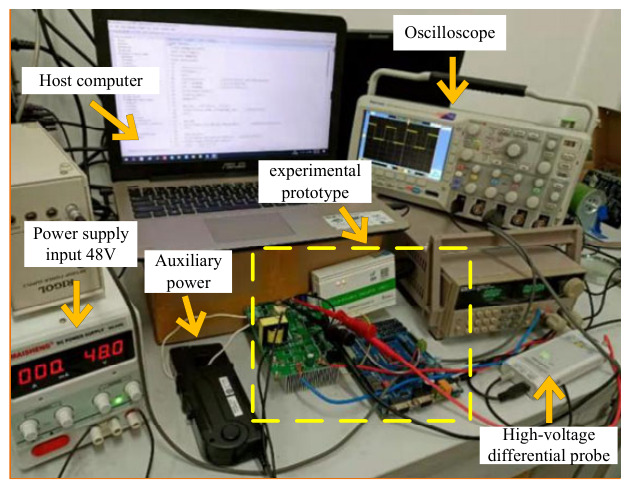
Experimental testing platform.

The current-sharing information between parallel modules is transmitted through a CAN communication network with a baud rate of 500 kbps. The experimental testing platform is illustrated in Figure 28.

**Figure 29:**
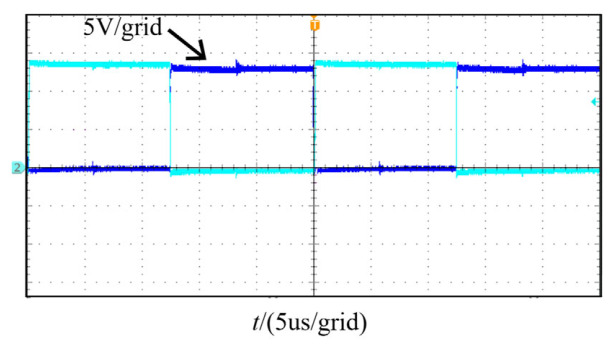
Driving waveform of the switch tube on the same axle arm.

**Figure 30:**
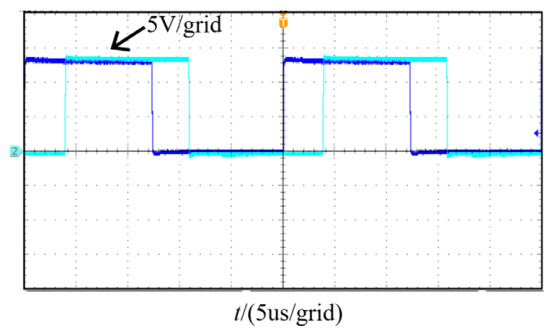
Driving waveforms of switches on different axle arms.

Figure 29 and Figure 30 present the PWM signal waveforms for two switches in the in-phase arm and the upper two switches in the out-of-phase arm, respectively. As the converter operates at a 40 kHz switching frequency, the switching cycle is 25 μs, which means each microsecond of time delay equals a phase shift of 14.4°. According to the oscilloscope measurement, the actual time delay Δt between cross-bridge driving signals is 40 μs, yielding an equivalent phase-shift angle of 216°. The measured equivalent shift angle verifies that the proposed phase-shift PWM strategy supports wide-range phase regulation, which fulfills the modulation requirement of the presented converter topology.

**Figure 31:**
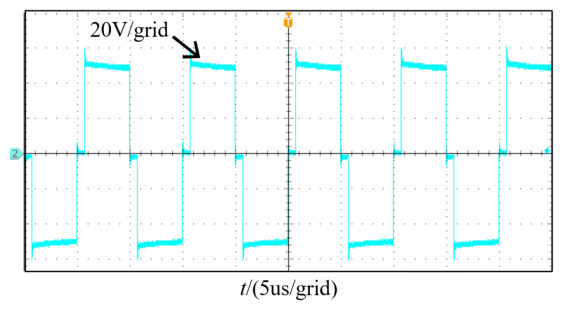
Output waveform of transformer primary side voltage.

**Figure 32:**
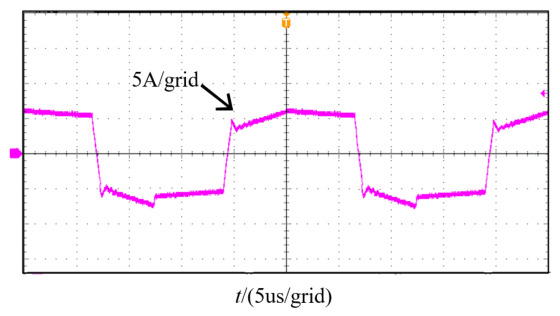
Output waveform of transformer primary side current.

Figure 31 presents the measured primary-side voltage waveform of the high-frequency transformer. A symmetrical square-wave voltage is observed across the transformer primary winding, with negligible voltage overshoot during the switching transitions. This demonstrates that the proposed converter achieves stable switching operation and provides a well-regulated excitation voltage for power transfer. As shown in Figure 32, the transformer primary current exhibits a typical trapezoidal waveform. The current increases linearly during the positive voltage interval and decreases linearly during the negative voltage interval, which is consistent with the voltage–current relationship of the leakage inductance.

**Figure 33:**
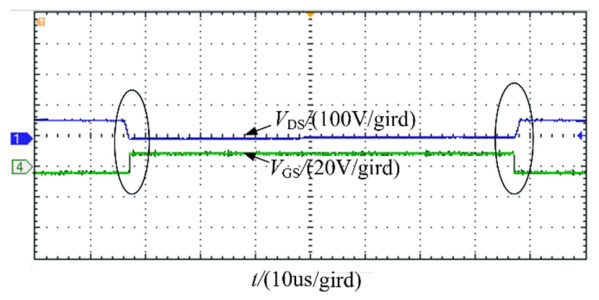
Detailed soft-switching waveform of the main switch Q1.

**Figure 34:**
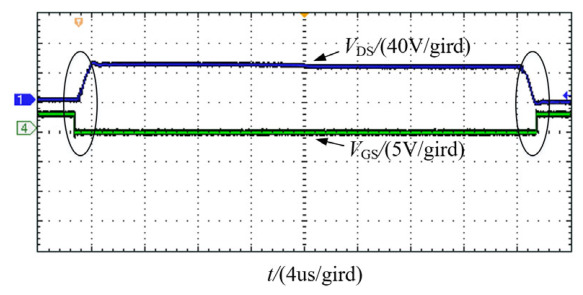
Detailed soft-switching waveform of the auxiliary switch Q3.

Figure 33 and Figure 34 present the detailed soft-switching waveforms of the main switch Q1 and the auxiliary switch Q3, respectively. As observed from the measured waveforms, the voltage across each switch approaches zero before the switching transitions occur, demonstrating successful zero-voltage switching operation. The experimental results validate the theoretical analysis and confirm the effectiveness of the proposed soft-switching mechanism.

**Figure 35:**
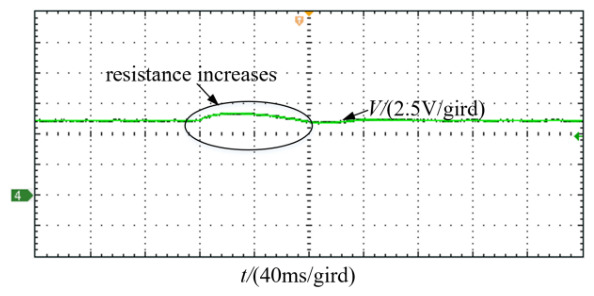
Output voltage waveform under light-load conditions.

**Figure 36:**
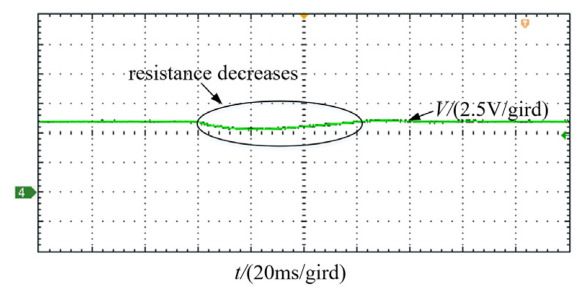
Output voltage waveform under heavy-load conditions.

During the experimental test, the electronic load was operated in the continuous dynamic mode, and the load resistance was periodically switched from 1 Ω to 0.75 Ω while the output voltage was regulated at 14 V. Figure 35 and Figure 36 illustrate the variations in the output voltage during load-resistance increase and decrease, respectively. As shown in Figure 33, the output voltage exhibits a maximum overshoot of 0.4 V and settles to its steady-state value within 80 ms following the load change. In Figure 34, the output voltage experiences a maximum undershoot of 0.3 V and recovers within 60 ms.

**Figure 37:**
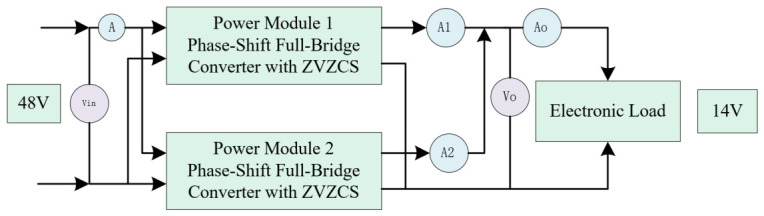
Block diagram of the output current test.

To evaluate the current sharing performance of the parallel LLSD-PSFB, we conducted relevant tests within the parallel system, following the output current testing framework illustrated in Figure 37.

The results of these tests are presented in Table 3. In parallel DC/DC converter systems, we typically assess the degree of current imbalance using the current sharing accuracy, denoted as CS_error_. The calculation formula for current sharing accuracy is provided in Equation (20).

(20) $$CS_{\mathrm{error}}=\Delta I_{0\max}/(I_{0}/N)$$

where ∆I_0max_ is defined as the maximum current deviation between any two operating modules in the parallel system, and Δ*I*0max denotes the maximum current deviation among all parallel modules. I_0_ represents the total output current of the paralleled system, and N denotes the number of modules operating in parallel.

To experimentally validate the current-sharing performance of the proposed control strategy for two paralleled power modules, load step tests are conducted at an output voltage of approximately 14 V. The system load is varied in steps to evaluate both steady-state and transient current-sharing characteristics. The branch currents, total output current, and current imbalance ratio are measured and recorded for analysis. The corresponding results are summarized in Table 3.

As shown in Table 3, the output currents of the two modules are well balanced throughout the tested load range. Based on the current-sharing error defined in (20), the current-sharing error remains below 5% in all cases, with a maximum value of 3.37% and a minimum value of 1.74% at full load. These results confirm that the proposed control strategy provides effective current sharing and satisfies the design requirements for parallel operation.

## 6. Conclusions

This paper proposes an LLSD-PSFB converter with potential applications in EV charging systems based on scalable parallel converter architectures. The operating principle, small-signal model, and parameter design procedure of the converter are analyzed. A three-loop current-sharing control strategy composed of a voltage loop, a current loop, and a current-sharing loop is developed for the parallel system. By regulating the difference between the module current and the average current through PI control, accurate current sharing among parallel modules is achieved.

Both simulation and experimental results demonstrate that the proposed control strategy achieves stable operation, fast dynamic response, and effective current-sharing performance. The results demonstrate the applicability of the proposed converter and control scheme for parallel EV charging systems. Future work will focus on higher-power applications and further studies on power losses, thermal performance, electromagnetic compatibility, and long-term reliability.

## Figures and Tables

**Table 1:** Main circuit parameters.

Parameter	Numerical Value	Parameter	Numerical Value
*V_in_*	48 V	*L_lk_*	3.3 µH
*K*	2	*C_r_*	22 nF
*C_b_*	10 µF	Output rectifier diode	MBR30200FCT
*L_f_*	0.8 µH	Lagging arm series diode	DESI30-06A
*C_f_*	220 µF		

**Table 2:** Parameters of PI controllers.

Control Loop	*K*p	*K*i	Zero Frequency
*G_CV_*	0.0479	18.05	60 Hz
*G_CC_*	0.3969	997.6	400 Hz
*G_CS_*	0.0576	8.68	24 Hz

**Table 3:** Test results of the output current for the parallel system.

Output Voltage	*I*_1_	*I*_2_	Total Current *I*_o_	Average Current	Imbalance
14.001 V	7095 mA	6915 mA	14,010 mA	7005 mA	2.57%
14.008 V	8190 mA	7920 mA	16,010 mA	8005 mA	3.37%
14.003 V	8930 mA	9180 mA	18,110 mA	9055 mA	2.76%
14.009 V	10,010 mA	10,120 mA	20,130 mA	10,065 mA	1.09%
14.005 V	10,910 mA	11,080 mA	21,990 mA	10,995 mA	1.55%
14.006 V	12,185 mA	11,975 mA	24,160 mA	12,080 mA	1.74%

## Data Availability

The original contributions presented in this study are included in the article. Further inquiries can be directed to the corresponding author.
